# Effect of monopolar capacitive resistive radiofrequency in treating stress urinary incontinence: A pilot randomized control trial

**DOI:** 10.3389/fpsyg.2022.1062363

**Published:** 2023-01-05

**Authors:** Eman A. Elhosary, Hamada Ahmed Hamada, Fatimah Ali AlMubali, Guillermo F. López Sánchez, Sara M. Ahmed

**Affiliations:** ^1^Department of Physical Therapy for Women Health, Faculty of Physical Therapy, Kafr Elshiekh University, Kafr Elshiekh, Egypt; ^2^Department of Biomechanics, Faculty of Physical Therapy, Cairo University, Cairo, Egypt; ^3^Department of Physical Therapy, Sharurah Armed Forces Hospital, Sharurah, Saudi Arabia; ^4^Division of Preventive Medicine and Public Health, Department of Public Health Sciences, School of Medicine, University of Murcia, Murcia, Spain; ^5^Department of Physical Therapy for Women’s Health, Cairo University, Giza, Egypt

**Keywords:** Stress urinary incontinence, 448 kHz monopolar capacitive resistive radiofrequency, pelvic floor exercises, the incontinence symptom severity index, perineometer

## Abstract

**Objective:**

To assess the effectiveness of 448 kHz monopolar capacitive resistive radiofrequency (MCRR) in the treatment of females with stress urinary incontinence (SUI).

**Materials and methods:**

Forty females with SUI complaints were separated randomly into two equal groups. Group A with 20 females received the MCRR therapy for 20 min and performed pelvic floor exercises for 20 min. Group B with 20 females received placebo treatment by applying the same application as in Group A without emitting any waves for 20 min, three times a week, for 4 weeks. The patients in both groups were instructed to pause the treatment during their menstruation; the patients were instructed to maintain home pelvic floor exercises. Both groups were assessed by a perineometer that was used to assess the strength of the pelvic floor muscles (PFM), the visual analogue scale (VAS), and the Incontinence Symptom Severity Index to assess the frequency of urinary incontinence symptoms as described by each patient before treatment and after 4 weeks of treatment.

**Results:**

There was a significant reduction (*p* < 0.05) in VAS and the Incontinence Symptom Severity Index and a significant increase in the strength of the PFM in both groups post-treatment compared with the pre-treatment. Regarding between-subject effects, there was a significant difference in VAS, the Incontinence Symptom Severity Index, and the perineometer between both groups (*p* < 0.05), and this significant improvement favored Group A.

**Conclusion:**

MCRR and pelvic floor exercises are more effective methods for the treatment of SUI than just pelvic floor exercises of females with SUI.

**Clinical Trial Registration:**

ClinicalTrials.gov. Identifier: NCT04612205.

## 1. Introduction

Stress urinary incontinence (SUI) is defined as the leak of a drop of urine that most commonly occurs with increased intra-abdominal pressure, resulting from coughing, laughing, or sneezing. It has a reflective psychosocial effect, which may affect a female’s well-being and her daily life, extending to her family and affecting the patient’s physical and social activities (William et al., 2021). Despite the fact that SUI is more common in older females, the main cause is not age; it can also occur in younger females, with an incidence of about 14% in women less than 30 years (Telma et al., 2020).

The main cause of SUI is the weakness or the straining of the pelvic floor muscles (PFM) resulting from pregnancy and vaginal delivery or nerve injuries to the lower back, which can lead to loss of bladder and urethral support and subsequent bladder leakage. The risk factors are obesity, chronic cough, and smoking which place frequent strain on the PFM (Vahdatpour et al., 2015).

Physical therapy modalities such as electrical stimulation, biofeedback, laser acupuncture, and vaginal cones have indicated effectiveness in the handling of such cases along with pelvic floor muscle exercises, but most females do not know how PFM works (Abdulaziz et al., 2022). Serotonin and noradrenaline reuptake inhibitors such as Duloxetine are utilized as a medical treatment for SUI. Due to drug interactions, Duloxetine, which was originally used as an antidepressant, should be used with caution in females who are also taking other antidepressant drugs (monoamine oxidase inhibitors, selective serotonin reuptake inhibitors). These medications, however, cause adverse effects such as tiredness, constipation, and dry mouth (Vella et al., 2008).

Radio waves are a physiotherapeutic form of electromagnetic waves, the mechanism of action is an electric current oscillation in the range of 2 to 3 thousand per second, converting it into heat released around the tip of the electrode that warms the medium (epidermis, dermis subcutaneous tissue, muscles, or joints). Radio waves increase circulation to the treated area, decreasing muscle tightness and producing a pain-relieving effect (Pihut et al., 2020). The thermal effect created in the irradiated tissue is due to the dilatation of blood vessels, the enhancement of tissue blood movement, the encouragement of metabolic and increase in tissue-absorption processes, growth in the number of leukocytes, decrease in muscle tension, decrease in excitability of the musculoskeletal-neural system, and the production of pain relief (Canpolat et al., 2018; Fang et al., 2018; Kwak et al., 2018). The abbreviation TECAR (Transfer Electrical, Capacitive, and Resistive) is originally used in Italy. TECAR therapy is a radiofrequency (RF) therapy, which produces high-frequency waves (Bretelle et al., 2020). RF can be capacitive or resistive (Kumaran and Watson, 2015). The capacitive mode (CET) generates energy in electrolyte-rich soft tissues such as muscles and vascular or lymphatic tissues (Navarro-Ledesma and Gonzalez-Muñoz, 2021). The resistive mode (RET) directs energy to deeper tissues that contain more fat and fibers (such as bones, ligaments, and tendons; Rodríguez-Sanz et al., 2022). High energy penetrates deep into the tissues, causing vascularization, analgesia, and a reduction in inflammation and edema, as well as helping the healing process (Weber and Kabelka, 2012; Duñabeitia et al., 2018).

SUI has a high prevalence (30%) in females of reproductive age, and 50% in those that are postmenopausal. Its effects on socio-psychological problems and have a significant effect on the economy. Regarding the effect of TECAR therapy in improving the strength and activity of the muscles (González-Gutiérrez et al., 2022). The goal of our research is to see how effective TECAR is in treating mild and moderate cases of females with SUI.

## 2. Materials and methods

### 2.1. Study design

This study was planned as a prospective, randomized, double-blind, pre–post-test, controlled trial. A physician referred all patients, after evaluation and approval of findings. This research was carried out in the Faculty of Physical Therapy’s outpatient clinic. All participants were told about the methods, and their free decision to participate was gained through informed consent. Each patient was given the option to withdraw from the trial at any time. Ethical approval was acquired from the Faculty of Physical Therapy before the study started (P.T. WH/3/2021/7), And has ClinicalTrials.gov Identifier: NCT04612205. The study was conducted between October 2020 and April 2021.

### 2.2. Participants

**Figure 1:**
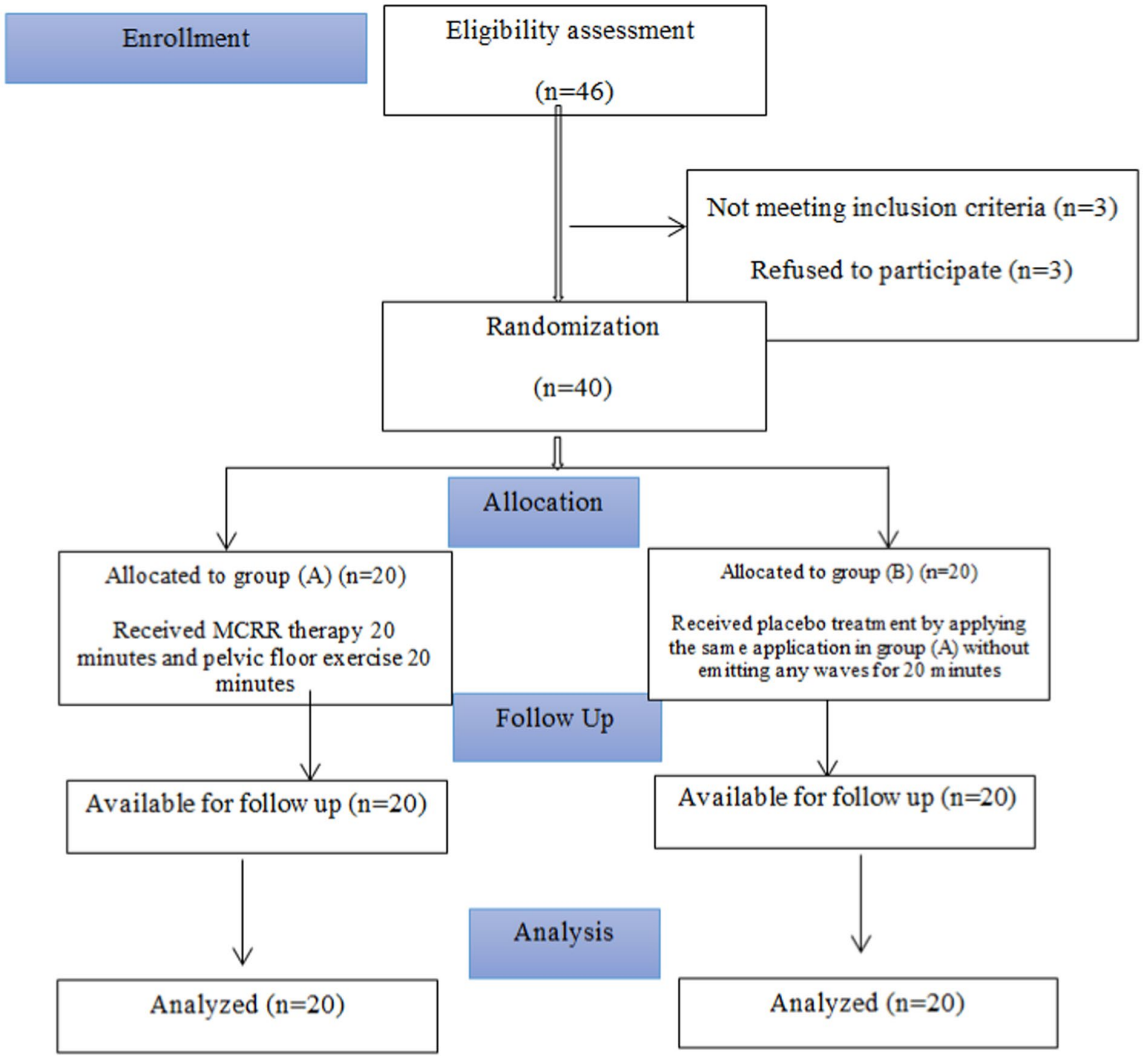
Flow chart of participants.

The participants included a sample of 40 females referred by a physician and diagnosed with mild and moderate SUI as assessed by the Incontinence Symptom Severity Index (Sandvik et al., 2000). Their ages ranged from 30 to 50 years. Following a brief explanation of the treatment procedure and the nature of the study, the randomization process was carried out through a computer program (Microsoft Excel 2016) that formed a random table of numbers in which each one was allocated to Group A or B (Figure 1). Patients were then matched according to the number conforming to their distribution code. A separate researcher directed the trials without informing patients and examiners to detect which patient was assigned to which group. Thus, both patients and evaluators were blinded to the treatment division. After randomization, no subjects withdrew from the study. The inclusion criteria were participants suffering from mild or moderate SUI due to pelvic floor weakness, hypermobility of the bladder neck, aged between 30 to 50 years, and body mass index from 30 to 32 kg/m2. Participants were excluded if they had diabetes, hypertension, cardiac diseases, bladder cancer, congenital urological disease, neuropathy and neurogenic bladder, detrusor hyperactivity, and bladder or urethra surgical interference. Participants were randomly allocated into both groups, Group A received the monopolar capacitive resistive radiofrequency (MCRR) therapy (the used device is described as monopolar by manufacturer) and performed pelvic floor exercises and Group B received placebo treatment and performed pelvic floor exercises. To avoid participants from suspect about the therapy to be placebo; the first time for all participants to receive TECAR therapy, no one of them can detect if device work or not, only seen that screen of device open and therapist work with device till time of treatment finish.

### 2.3. Instruments

A perineometer was used for evaluating the strength of the pelvic floor before treatment and 4 weeks after treatment (Peritron 9300): Cardio design Peritron 9,300 in Australia. It has a vaginal sensor with a diameter of 28 mm. Mechanical description: Numerical readout range from 0 to 300 cm H₂O. It was used before treatment and after 12 sessions, as an objective evaluation of the strength of the PFM and as muscle re-education (Abe-Takahashi et al., 2020).

The Incontinence Symptom Severity Index is a tool used for evaluating female patients with frequent urinary evacuation symptoms. It is used as an assessment tool and for detecting feedback to interferences. The index rate acquired by multiplying the total amounts in the two parameters (point for frequency and point of amount), is further characterized into a severity index of three or four levels. The score ranges from 0 to 12. A high score indicates incontinence severity. The score uses two questions: “How often is urine leakage experienced?” (Never = 0, Less than once a month =1, One to several times a month = 2, One to several times a week = 3, Every day and/or night = 4); “How much urine is lost each time?” (A few drops = 1, A little = 2, More = 3). Severity index = (points for frequency) × (points for amount); 1–2 = slight, 3–6 = moderate, 8–9 = severe, 12 = very severe (Sandvik et al., 2000; Christian et al., 2009; Nacif et al., 2022).

The visual analogue scale (VAS) is a graphic evaluation tool used by patients for measuring the severity of stress incontinence symptoms. Each patient places her estimation on VAS with numerical rates placed equally along a 10 cm line. The signifiers and numbers aid the patient to place her estimation on the line. Both groups of patients use the scale before and after the treatment (Hartrick et al., 2003). TECAR device: MCRR. The sign producer is an INDIBA® 448 kHz RF device that has a vaginal/ or rectum electrode (made in Barcelona, Spain).

### 2.4. Procedure

In Group A, the patient was asked to lie down in the crock lying position with knees apart and supported, and the patient was asked to relax. The women’s physical therapy specialist applied a session of MCRR followed by pelvic floor exercises and taught every patient the home routine. MCRR used RF in the resistive mode. The therapist used a lubrication gel in the vagina and introduced the electrode after sterilization for 20 min per session at 1–5% of the power range, sub-thermal level. The treatment included 12 sessions of RF, 3 times per week for 4 weeks. During the application of MCRR, each patient contracted the PFM and avoided contraction of the abdomen, glutei, and hip adductor muscles. Patients were asked to contract as if she control her bowel action, urethral orifice, concentrate in this action, perform contractions of the muscles 20 times as strong as they can for 10 s and then relax for 20 s followed by rest for 2 min. The exercises were performed for 20 min after MCRR application, 3 times per week, and for 4 successive weeks. To prevent urine leakage, Knack technique was taught as the patients were instructed to contract their PFM before coughing or sneezing. Such contraction elevates the pelvic floor cranially, with consequent closure of the urethra, vagina, and rectum. The patients were asked to make exercises a home routine, in bed, morning and night in the crock lying position, and in sitting and standing positions during the day.

The women in the control group received a placebo treatment, which was conducted by applying the same treatment as in Group A but without emitting waves. Each participant was then instructed to perform pelvic floor exercises. The exercise program lasted for 20 min after the application of MCRR placebo treatment, 3 times a week for 4 weeks in addition to exercises at home as Group A. The patients in both groups were instructed to pause treatment during menstruation.

The primary outcome measure was the strength of the PFM as measured by the perineometer before and after 4 weeks of treatment. SUI severity, as determined by the urine incontinence severity index and VAS before and after 15 min of rest after the end of last session of treatment, was the secondary outcome.

### 2.5. Statistical analysis

**Figure 2:**
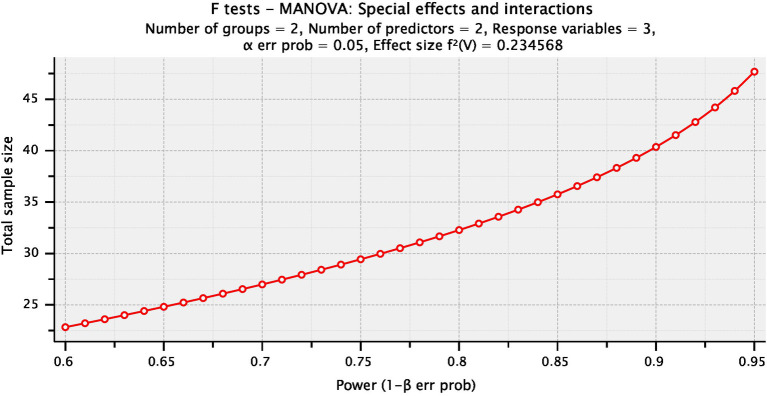
Plot of sample size calculation.

The sample size was determined by estimating the trial size before the survey using the statistical programming software G*POWER (version 3.1.9.2; Franz Faul, Universitat Kiel, Germany) [F tests-MANOVA: Special effects and interaction, α = 0.05, β = 0.20, number of predictors = 2, number of dependents = **3**, Pillai V = 0.**19**, and effect size = 0.**23**] and revealed that the appropriate sample size for this study was N = 3**3**]. The magnitude of the effects was determined for the pilot trial with 10 participants (5 per group; Figure 2). The statistical analysis was carried out with the help of Windows SPSS version 23 (SPSS, Inc., Chicago, IL). For comparing the variables of interest examined in both groups, a 2 × 2 mixed-design MANOVA was used. The data were analyzed for the normality hypothesis, variance homogeneity, and the occurrence of extreme scores. This scan was performed as a preliminary condition for the difference analysis parameter computations. The value of *p* has been set at 0.05.

## 3. Results

The overall characteristics of all subjects in the two groups (A and B) at the beginning of the study are presented in Tables 1, 2. In the average comparison of all measured parameters and both groups, the inferential statistics revealed no significance.

**Table 1:** General characteristics of both groups.

Variables	Group A (*n* = 20)	Group B (*n* = 20)	*t*-value	*p*-value
Age (years)	42.35 ± 4.73	44.35 ± 4.73	−1.335	0.19
BMI (Kg/m^2^)	29.6 ± 0.68	29.5 ± 1.14	0.335	0.74

Significance is considered at *p* < 0.05.

**Table 2:** Clinical data of the Studied Groups (A and B).

Clinical data	Group A (*N* = 20)		Group B (*N* = 20)		χ^2^	*p*-value	N	%	N	%
Parity					1.029	0.598
Multi	18	90	17	85		
Null	0	0	1	5		
Primi	2	10	2	10		
Occupation					0.173	0.677
Work	4	20	3	15		
Not work	16	80	17	85		
Level of education					2.437	0.296
Basic	11	55	14	70		
High	7	35	6	30		
Read and write only	2	10	0	0		

χ^2^: Chi-squared value.

Tables 3, 4 show descriptive data, 95% CI differences in the effects of VAS, perineometer, and The incontinence symptom severity index within and across groups. In the same context, the multiple pairwise comparison tests revealed that there was a significant reduction (p 0.05) in VAS and The incontinence symptom severity index and a significant increase in the perineometer in both groups in the post-treatment condition compared to the pre-treatment condition in both groups. Regarding between-subject effects, multiple pairwise comparisons revealed that there was a significant difference in VAS, perineometer, and The incontinence symptom severity index between both groups (*p* < 0.05) and this significant improvement favors Group A.

**Table 3:** Descriptive statistics for all VAS, the incontinence symptom severity index and Periniomter for both groups at different training periods.

Variables	Group (A)			Group (B)			Pre	Post	*p*-value (effect size)	Pre	Post	*p*-value (effect size)
Periniomter	35.65 ± 9.32	79.1 ± 6.69	0.0001 (4.66)	32.35 ± 8.7	57.7 ± 5.69	0.0001 (2.91)
VAS	7.55 ± 0.82	0.65 ± 0.6	0.0001 (8.41)	7.45 ± 0.99	4.1 ± 1.44	0.0001 (3.38)
The incontinence symptom severity index	4.65 ± 1.18	0.35 ± 0.4	0.0001 (3.64)	4.85 ± 1.26	1.85 ± 0.87	0.0001 (2.38)

Values of all dependent variables are expressed as mean ± SD, VAS: Visual Analogue Scale.

**Table 4:** Within and between groups differences at 95% CI for the effects of interventions.

Variables	Within groups		Between groups	
	Group (A)	Group (B)	Mean difference (95% CI)	MCID
	Mean difference (95% CI)	Mean difference (95% CI)		
Periniomter	−43.45 (−48.253 to-38.647)*	−25.35 (−30.153 to-20.547)*	−18.1 (−20.45 to-15.75)*	3.32
VAS	6.9 (6.523 to 7.277)*	3.35 (2.973 to 3.727) *	3.55 (3.26 to 3.84)*	0.41
The incontinence symptom severity index	4.3 (3.651 to 4.949)*	3 (2.351 to 3.649) *	1.3 (−1.01 to 1.58)*	0.406

CI: Confidence Interval, MCID: Minimal Clinically Important Difference, VAS: Visual Analogue Scale.

* The mean difference is significant at the 0.05 level.

## 4. Discussion

Urinary incontinence is a health issue that disturbs the quality of life (QOL) for women at different phases of life. SUI can be attributed to vaginal deliveries and estrogen deficiency in the perimenopausal period. The most commonly suggested first-choice therapy approach is Kegel exercises or pelvic floor exercises (Ptak et al., 2019; Frawley et al., 2021).

The current study’s findings show that both groups’ pelvic floor strength and SUI symptoms improved significantly in the following therapy as compared to pre-treatment levels. The results also show statistically significant higher values of pelvic floor strength and SUI symptoms for Group A after treatment compared to Group B. These results are consistent with Dayan et al. (2019) study, which showed that maximal pelvic muscle contraction (UROstym max) measurements were enhanced after initial treatment by RF. This may be clarified by the motor-unit recruitment phenomenon, that is, the stimulation of further motor units for the augmented contractile strength in a muscle (Lalji and Lozanova, 2017). RF is a more advanced therapeutic device for treating SUI, and it has gained significant popularity lately as a result of its non-invasiveness, lack of side effects, and fast outcomes. RF is based on increasing the heat of the target tissue to stimulate biological changes; the RF-produced heat induces the tissue matrix of collagen, elastin, and ground substances and stimulates instantaneous variation in the helical structure of the collagen (Allan et al., 2020a). In addition, neocollagenesis and neoelastogenesis are activated as a result of the micro-inflammatory stimulation of fibroblasts. Furthermore, the synthesis of sex steroid precursor dehydroepiandrosterone (DHEA) is triggered. Estrogen synthesis is enhanced by DHEA in the vulvo-vaginal tissue, which is responsible for revitalizing and promoting the vaginal tissue and collagen (Garzon et al., 2021). Moreover, the recognized tightening outcome of RF energy recovers the muscle length/tension connection, permitting increased contractile efficacy as defined in the Frank-Starling relationship. Improvements in stress incontinence, atrophic vaginitis, and orgasmic dysfunction, especially after initial RF treatment, with some further improvement observed after the second and third treatments, were noted by Stachowicz et al. (2021).

Consistent with our findings, Lordêlo et al. (2017), who studied the effect of RF on SUI, showed that SUI improved in seven out of 10 participants. These results may be due to increased blood supply and opening of vascular tissues occurring as a result of heating. This phenomenon results in the improvement of the venous plexus (Fleischmann et al., 2003). The observed improvement could be attributed to the time of collagen denaturation and neo-synthesis, which lasts around 28 days after therapy. The mechanism of urethral closure will improve when the collagen changes (Mezzana et al., 2022). Rechberger et al. (1998) also demonstrated that there is a strong correlation between collagen content, urethral pressure, the length of the urethra, and the maximum closure pressure of the urethra.

The results of the study are consistent with those of Lalji and Lozanova (2017) who confirmed that the monopolar RF intervention is considered an effective and safe therapy for SUI. Allan et al. (2020b) also reported that SUI symptoms as judged by SUI-reported outcomes and the objective one-hour pad weight test, with a > 50% drop in pad weight from baseline for 52% of the patients at 12 months. Tashiro et al. (2017) stated that TECAR therapy can stimulate a deep-tissue response without an unwanted elevation of skin temperature, which is an amazing advantage for a new intervention with a thermotherapeutic effect.

Szabo et al. (2020) stated that the recovery protocol of combining therapeutic physical exercises with TECAR therapy has beneficial outcomes in the rehabilitation process. In addition, Kim et al. (2020) explained that TECAR therapy has a programmed mode, making it possible to carry out training while applying high-frequency current. This highlights the results of our study that the intracavitary TECAR probe provides strong sensory feedback for performing Kegel exercises while transmitting high-frequency current to the pelvic floor muscle. Hawamdeh (2014) concluded that TECAR therapy helps reduce recovery time with a series of repetitive muscle contractions.

The findings of this study are consistent with those of Bo (2004) and Hay Smith et al. (2009). Both studies found that Kegel exercises are effective in a variety of ways: women were taught to pre-contract the pelvic floor muscles before and during increases in intra-abdominal pressure to avoid urine leakage, and strength exercises reinforced lifelong muscle volume and thus provided mechanical support. In addition, Mahmoud et al. (2018) concluded that the duration of the treatment with Kegel exercises should not be less than 6 weeks. It also strengthens pelvic floor muscles, minimizes SUI symptoms, and consequently improves the QOL. Pelvis floor training improves resting muscle tone, boosts the number of recruited motor units, and triggers muscle growth. Pelvic floor training directly increases muscle strength, which influences the support function, the time of maximum contraction, deep sensitivity, and the enhancement of neuromuscular control (Namysł, 2003; Santiagu, 2008). Bo et al. (1999) found that for SUI, pelvic floor training was more efficient than electrotherapy, vaginal cones, and no treatment control. The results of this study are also consistent with that of Cavkaytar et al. (2015) that stated that with PFE there is a speed up in recovery of 68.4% of the women complaining from SUI and 41.2% of the women diagnosed with mixed urinary incontinence (MUI), they stated that a home program of Kegels exercises with no guidance is helpful in women with SUI and MUI and the recovery is significant in women with SUI. Consistent with our findings, Soni et al. (2014) demonstrated that the detected increase in strength and endurance of pelvic floor muscles was related to Kegel exercise training. Endurance improvement refers to the improved holding capability with less or no incident of leakage. Radzimińska et al. (2018) stated that pelvic floor muscle training (PFMT) is a successful, non-invasive, and first conservative approach for women. The protocol of PFMT must continue for at least 6 weeks. PFMT should be conducted under supervision. It can be used as a standalone or in combination with other treatment modalities in females with SUI. Jacomo et al. (2020) also stated that PFMT remains the cornerstone technique for augmenting pelvic muscle strength.

The limitations of this study may be the lack of follow-up on the effectiveness of 448 kHz MCRR in the treatment of females with SUI complaints for several months after rehabilitation. Participants belonged to a wide age group and had different educational levels. Accordingly, it can be concluded that 448 kHz RF intervention is an efficient, safe, and successful therapy in combination with Kegel exercises in treating SUI.

## 5. Conclusion

MCRR and pelvic floor exercises are more effective methods for the treatment of SUI than just pelvic floor exercises of females with SUI.

## Data availability statement

The raw data supporting the conclusions of this article will be made available by the authors, without undue reservation.

## Ethics statement

The studies involving human participants were reviewed and approved by Faculty of Physical Therapy, Kafr Elshiekh University. The patients/participants provided their written informed consent to participate in this study. Ethical approval number (P.T. WH/3/2021/7).

## Author contributions

All the authors have made substantial contributions to conception and design, collection of data, or analysis of data, and have been involved in drafting the manuscript or revising it critically for important intellectual content. All the authors have read and approved the final manuscript.

## Conflict of interest

The authors declare that the research was conducted in the absence of any commercial or financial relationships that could be construed as a potential conflict of interest.

## Publisher’s note

All claims expressed in this article are solely those of the authors and do not necessarily represent those of their affiliated organizations, or those of the publisher, the editors and the reviewers. Any product that may be evaluated in this article, or claim that may be made by its manufacturer, is not guaranteed or endorsed by the publisher.
